# Comparison of overfeeding effects on gut physiology and microbiota in two goose breeds

**DOI:** 10.1016/j.psj.2020.12.057

**Published:** 2020-12-24

**Authors:** R.X. Wei, F.J. Ye, F. He, Q. Song, X.P. Xiong, W.L. Yang, X. Gang, J.W. Hu, B. Hu, H.Y. Xu, L. Li, H.H. Liu, X.Y. Zeng, L. Chen, B. Kang, C.C. Han

**Affiliations:** ∗ Farm Animal Genetic Resources Exploration and Innovation Key Laboratory of Sichuan Province, Sichuan Agricultural University, Chengdu, Sichuan 611130, P.R. China; † College of Life Science, Sichuan Agricultural University, Ya'an, Sichuan 625014, P.R. China; ‡ Xichang Huanong Poultry Co., Xichang, Sichuan 615000, P.R. China

**Keywords:** overfeeding, ERIC-PCR, antioxidant capacity, intestinal physiology, enteric microorganism

## Abstract

To have a better understanding of how the “gut–liver axis” mediates the lipid deposition in the liver, a comparison of overfeeding influence on intestine physiology and microbiota between Gang Goose and Tianfu Meat Goose was performed in this study. After force-feeding, compared with Gang Goose, Tianfu Meat Goose had better fat storage capacity in liver (397.94 vs. 166.54 for *foie gras* weight (g), *P* < 0.05; 6.37 vs. 2.92% for the ratio of liver to body, *P* < 0.05; 60.01 vs. 46.64% for fat content, *P* < 0.05) and the less subcutaneous adipose tissue weight (1240.96 g vs. 1440.46 g, *P* < 0.05). After force-feeding, the digestion–absorption capacity of Tianfu Meat Goose was higher than that of Gang Goose (5.56 vs. 3.64 and 4.63 vs. 3.68 for the ratio of villus height to crypt depth in duodenum and ileum, respectively, *P* < 0.05; 1394.96 vs. 782.59 and 1314.76 vs. 766.17 for the invertase activity (U/mg-prot), in duodenum and ileum, respectively, *P* < 0.05; 6038.36 vs. 3088.29 and 4645.29 vs. 3927.61 for the activity of maltase (U/mg-prot), in duodenum and ileum, respectively, *P* < 0.05). Force-feeding decreased the gene expression of *Escherichia coli* in the ileum of Tianfu Meat Goose; force-feeding increased the number of gut microbiota Enterobacterial Repetitive Intergenic Consensus-Polymerase Chain Reaction band in Tianfu Meat Goose and decreased the number in Gang Goose. In conclusion, compared with Gang Goose, the lipid deposition in the liver and the intestine digestion–absorption capacity and stability were higher in Tianfu Meat Goose. Thereby, Tianfu Meat Goose is the better breed for *foie gras* production for prolonged force-feeding; Gang Goose possesses better fat storage capacity in subcutaneous adipose tissue. However, Gang Goose has lower gut stability responding to force-feeding, so Gang Goose is suited to force-feeding in a short time to gain the body weight and subcutaneous fat as an overfed duck for roast duck.

## Introduction

The liver is the most important metabolic organ in the body. The intestine is the main place where nutrient digestive absorption takes place, and gut flora colonizes, which plays an important role in the growth and metabolism of organisms. The liver and gastrointestinal tract are closely related in anatomy and function, forming the whole digestive system. Since the theory of “the gut–liver axis” (Miura and Ohnishi, 2014) was suggested, the relationship between liver and intestine has attracted a great deal of attention for disease research. There is increasing evidence that the occurrence of nonalcoholic fatty liver disease (**NAFLD**) in mammals is closely associated with the intestinal environment (Bajaj and Hylemon, 2018).

The intestine plays an important role in the digestion, absorption, and transportation of nutrients. Mitchell and Smith studied 3 broiler strains with different growth rates; the result showed that the fastest growing strain had the highest absolute intestinal weight and length (Mitchell and Smith, 1991). The integrity of intestinal mucosa morphology is the basis of maintaining normal intestinal physiological activity in animals (Houshmand et al., 2012). The higher ratio of villus height to crypt depth reflects higher nutrient absorption capacity (Liu et al., 2010). The digestive enzymes in the intestinal tract not only decompose food into small molecules but also produce a variety of oxidizing factors, such as iron, copper, aldehyde, lipid peroxide, and so on, which makes the intestinal tract more vulnerable to free radicals (Kanner and Lapidot, 2001). The occurrence of oxidative stress, in return, will decrease the activity of digestive enzymes and damage the integrity of intestinal mucosa morphology. Some researchers reported that excessive feed input in waterfowls negatively affected nutrients digestibility, absorption, and utilization (Zhang et al., 2007). However, there have seldom been reports about the influence of force-feeding on intestine physiology in waterfowls.

A large number of microorganisms colonize the intestinal tract of humans and animals. More and more evidence suggested that gut microbiota regulated the goose fatty liver formation in the force-feeding process. Lactic acid is known as the main metabolite of intestinal *Lactobacillus*. The content of lactic acid in the serum and the intestine in the overfed geese is significantly higher than that in the control group. Therefore, it is speculated that lactic acid could have a protective effect against the formation of goose fatty liver (Liu et al., 2016). In overfed goose, the complement system that mediates inflammation was suppressed because of the increasing level of blood lactic acid produced by the enriched *Lactobacillus*, and *TNFα* was suppressed by the lactic acid via HNF1*α*/C5 pathway (Liu et al., 2016). Different genotypes of goose have a different composition of intestinal microbiota. For the moment, researches involved in influence of force-feeding on gut microbial community mainly focus on Langdes Goose and have been seldom reported in other goose breeds.

China has the largest number of goose breeds, including indigenous goose breeds and developed goose breeds in the world. Gang Goose is an excellent indigenous goose breed distributing in southwest China. It has the characteristics of large body size, fast growth speed, and strong fat storage capacity. Tianfu Meat Goose is a developed goose breed from the Sichuan Agricultural University with many outstanding performances, such as excellent egg-laying performance, fast-growth speed, and strong adaptability (Chen et al., 2004). The main purpose of force-feeding is to increase body fat deposition and produce *foie gras* in waterfowl. Force-feeding can cause different influences on the gut physiology and gut flora in different goose breeds, and different goose breeds have different production performance of *foie gras*. To understand more comprehensively how these gut physiology and flora differences lead to different lipid deposition regulation pattern caused by force-feeding, Gang Goose and Tianfu Meat Goose were taken as the research object in this study, and the difference in the influence of force-feeding on these 2 goose breeds was compared from intestine physiology and enteric microorganism. Goose fatty liver is similar to the nonalcoholic fatty liver; *foie gras* presents a unique model of hepatic steatosis (Geng et al., 2016). Not only will understanding these difference mechanisms provide ideas for the development and utilization of breed resources for *foie gras*, it is also conducive to a greater understanding of the “gut–liver axis”. Meanwhile, it will provide not only a reference for exploring the relationship between intestinal health and the mechanism of goose fatty liver formation but also an approach to the prevention and treatment of fatty liver disease in humans.

## Materials and methods

### Ethics Statement

All procedures in the present study were subject to approval by the Institutional Animal Care and Use Committee of the Sichuan Agricultural University (Permit No. DKY-B20141401) and carried out in accordance with the approved guidelines. All efforts were made to minimize the suffering of the animals. The movement of birds was not restricted before the age of 90 d. The experimental geese were killed with an electrolethaler before harvesting their liver samples and intestinal contents.

### Birds and Experiment Design

This trial was carried out at the Xichang Huanong Poultry Co. (Sichuan, China). A total of 55 healthy 90-day-old Gang Geese and Tianfu Meat Geese were separated respectively into normal feeding and force-fed groups randomly (n = 15 and 40, respectively). The geese of the force-fed group were force-fed with boiled maize (maize boiled for 5 min, supplemented with 1% plant oil and 1% salt), and the daily feed intake reached 600 to 750 g (4 meals a day), which lasted 3 wk; the geese in the normal feeding group were allowed ad libitum access to diet. Birds had free access to water at all times. The routine husbandry management was carried out through the experiments.

### Sampling, Slaughter Performance Analysis, and Liver Quality Determination

Birds were weighed individually at 111 d (before slaughter) after 24 h of fasting. Carcasses were eviscerated 20 min postmortem after scalding and plucking. The samples of abdominal fat and subcutaneous adipose tissue were collected and weighed immediately. After carcass evisceration, the liver was removed and weighed. The sampling of *foie gras* was carried out as described by Fernandez et al. (Fernandez et al., 2011). The weight and length of each intestine section were measured and weighted after slaughter and division. A 30 ± 10 g sample of the liver was taken from the central part of the large lobe. This sample was immediately frozen and stored at −20°C until chemical analysis. The water content of the liver was determined by the constant temperature drying method in a dry box, and the crude fat content was determined by the Soxhlet leaching method (Zhang, 2016).

### Sampling and Histomorphological Examinations for Liver and Small Intestine

Five geese of each group were killed and then immediately sacrificed for liver and small intestinal tissue. Liver and small intestinal tissue were washed in ice-cold saline (0.9% NaCl; 4°C) and fixed in 4% formaldehyde-phosphate buffer for histomorphology determination. According to the methods of Hou et al. (Hou et al., 2020), the cross-sections from the middle of the duodenum, jejunum, ileum, and liver preserved in 4% formaldehyde-phosphate buffer were prepared using standard paraffin embedding techniques, sectioned (5 μm) and stained with hematoxylin and eosin, and sealed by neutral resin size thereafter and then examined by microscope photography system (Olympus, Tokyo, Japan). Each slice was observed, and 5 visual fields were randomly selected at 20 × magnifications. The selected visual fields were measured via imaging software (Image Pro Plus 6.0, Media Cybernetics, Bethesda, MD). The visual measurements of the villus height, crypt depth, intestinal wall thickness, and liver fat droplet area ratio were measured 10 times, and an average was taken.

### Sampling, Enzymatic Determination, and Enteric Microorganism Analysis

When the small intestinal tissue samples used for histotomy were collected, the small intestinal contents of each intestinal section were collected, frozen in liquid nitrogen immediately, and then kept at −80°C for digestive enzyme activity, antioxidant capacity, Enterobacterial Repetitive Intergenic Consensus-Polymerase Chain Reaction (ERIC-PCR), and fluorescence quantitative PCR of gut flora assay.

The protein concentration of the samples was employed to calculate enzyme activity. The protein concentration of samples was employed to calculate the digestive activities and antioxidant capacity and assayed using a protein quantification kit (Bicinchoninic Acid Assay, Beyotime Biotechnology, Beijing, China); the kits that assayed the activity of amylase, maltase, invertase, chymotrypsin, total antioxidant capacity (**T-AOC**), total glutathione peroxidase (**TGP**), superoxide dismutase (**SOD**), catalase (**CAT**), and malonyldialdehyde (**MAD**) were provided from the Nanjing Jiancheng Bioengineering Institute (Nanjing, China). All operations were carried out according to the directions. Each test included 5 biological samples, and each sample was analyzed in triplicate.

The bacterial genomic DNA from small intestinal contents extraction was extracted according to the fecal DNA extraction kit (DP328, Tiangen, Beijing, China). The extracted DNA was determined by ultraviolet spectrophotometer (Nano Drop 2000, Thermo Fisher Scientific, Waltham, MA). The purity and quality of total DNA were assessed by spectrophotometric absorbance at 260/280 nm, and the integrity of DNA was detected by agarose gel electrophoresis, and the results were observed and photographed in the gel imaging system (Bio-Rad, Hercules, CA).

The fluorescence quantitative PCR was performed on the CFX 96 instrument (Bio-Rad), using a Takara ExTaq RT-PCR kit and SYBR Green as the detection dye (Takara). The qRT-PCR reaction system contained the sample DNA template (1.0 μL), SYBR Premix Ex Taq TM (6.0 μL), sterile water (4.0 μL), upstream primers of target genes (0.5 μL), and downstream primers of target genes (0.5 μL). After initial denaturation at 95°C for 5 min, 40 cycles were carried out: 95°C for 10 s, 60°C for 20 s, 72°C for 15 s, and 72°C extensions for 10 min. Supplementary Table 1 listed the fluorescence quantitative PCR primers (BGI, Beijing, China). The relative gene expression levels of genes were normalized to *β-actin* and *18S* using the 2^−ΔΔCt^ method (Livak and Schmittgen, 2001). Exp _target gene in sample_ = (1 + R_target gene_) ^Ct (target gene in sample)^/(1 + R_β-actin_ _or 18S_) ^Ct^ (β-actin ^or 18S in sample)^. Each test included 5 biological samples, and each sample was analyzed in triplicate.

The total DNA extracted from small intestinal bacteria was used to perform ERIC-PCR. Primers were synthesized by BGI. Primer sequences were shown as below: ERIC-1: 5′-ATGTAAGCTCCTGGGGATTCAA-3'; ERIC-2: 5′-AAGTAAGTGACTGGGGTGAGCG-3' (Wang et al., 2014). The ERIC-PCR assay was performed as per the method described by Staji et al. (Staji et al., 2018). ERIC-PCR amplication reactions were performed in 25 μL volume containing 1.5 μL of each primer (25 pM), 2 μL of 2.5 mM dNTPs, 2.5 μL of 10 × buffer, 2 μL of 25 mM MgCl_2_, 0.5 μL of 5 U/μL Taq Polymerase (Sigma Aldrich, Shanghai, China), and 10 ng of template DNA. After initially denaturation at 94°C for 7 min, 30 cycles were carried out: 94°C for 60 s, 52°C for 60 s, 65°C for 8 min, and 65°C extensions 10 min. The ERIC-PCR products were isolated by electrophoresis (100 V, 30 min) in agarose gel (1.5%), the marker used was DNA marker DL5000 (Takara), and the electrophoresis results were observed and photographed in the gel imaging system (Bio-Rad).

### Statistical Analysis

By using SAS 9.13 package (SAS Institute Inc, Cary, NC), the comparisons of multiple groups were analyzed by GLM, and the means were assessed for significant differences using the SNK-q test. All data were presented as means ± SD and showed with graphs created with GraphPad Prism 8.0 software (GraphPad Prism Software, Inc.). *P* < 0.05 was considered statistically significant. The gel imaging system was used to take pictures of the stained gel, and Quantity One image analysis software (Bio-Rad) was used for ERIC-PCR image analysis.

## Results

### Comparison of Force-Feeding Influence on the Slaughter Performance and Liver Lipid Deposition in 2 Goose Breeds

**Figure 1:**
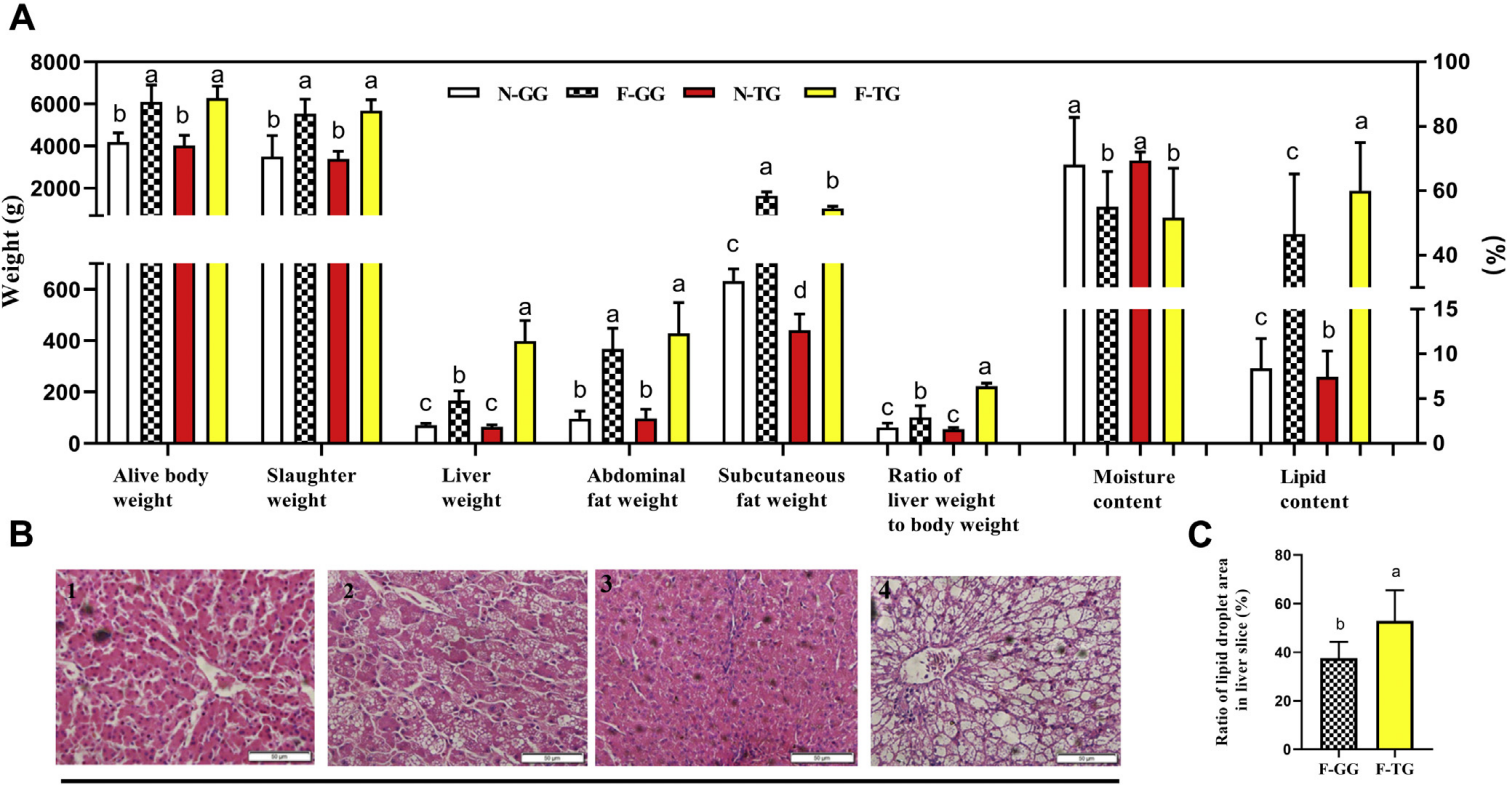
Comparison of force-feeding influence between 2 breeds on the slaughter performance and liver lipid deposition. (A) Force-feeding influence on the slaughter performance; normal-feeding group n = 15 birds, force-feeding group n = 40 birds. (B) Force-feeding influence on liver morphology (n = 5); 1, liver slice of N-GG; 2, liver slice of F-GG; 3, liver slice of N-TG; 4, liver slice of F-TG. (C) Ratio of fat droplet area in liver slice (n = 5). The experimental values are the means ± SD. The different lowercase above the bars represent significant differences (*P* < 0.05). Abbreviations: F-GG, force-feeding group of Gang Goose; F-TG, force-feeding group of Tianfu Meat Goose; N-GG, normal-feeding group of Gang Goose; N-TG, normal-feeding group of Tianfu Meat Goose.

Compared with Gang Goose, the liver weight, the crude fat content of the liver, and the ratio of the liver was higher (*P* < 0.05) after overfeeding in Tianfu Meat Goose (Figure 1A). Force-feeding induced more serious steatosis in the liver of Tianfu Meat Goose (Figures 1B and 1C; Supplementary Figure 1). The subcutaneous adipose tissue weight of Gang Goose was higher than that of Tianfu Meat Goose after overfeeding (*P* < 0.05) (Figure 1A). There was no difference in the abdominal fat weight (*P* > 0.05) (Figure 1A).

### Comparison of Force-Feeding Influence on the Intestinal Morphology in 2 Goose Breeds

**Figure 2:**
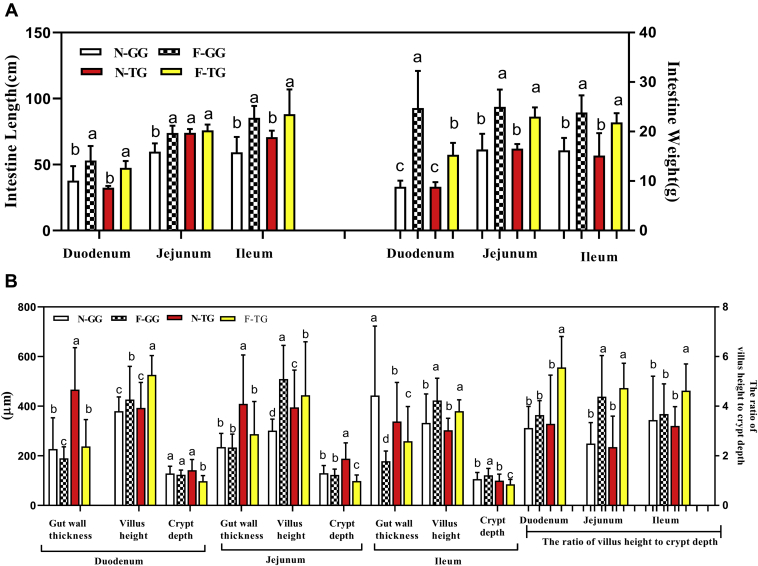
Comparison of force-feeding influence between 2 breeds on small intestinal histology. (A) Force-feeding influence on small intestine length and weight; normal-feeding group n = 15 birds, force-fed group n = 40 birds. (B) Force-feeding influence on intestine slice index (n = 5). The experimental values are the means ± SD. The different lowercase above the bars represent significant differences (*P* < 0.05). Abbreviations: F-GG, force-feeding group of Gang Goose; F-TG, force-feeding group of Tianfu Meat Goose; N-GG, normal-feeding group of Gang Goose; N-TG, normal-feeding group of Tianfu Meat Goose.

Force-feeding increased the small intestinal length and weight of 2 breeds (*P* < 0.05) (Figure 2A). Supplementary figures (Supplementary Figures 2–4) showed the intestinal tissue slice image of duodenum, jejunum, and ileum, respectively. As shown in Figure 2B, force-feeding decreased the intestinal wall thickness (*P* < 0.05) and increased the intestinal villus height (*P* < 0.05) in both breeds, and force-feeding decreased the small intestinal crypt depth of Tianfu Meat Goose (*P* < 0.05). Compared with Gang Goose, the ratio of villus height to crypt depth of duodenum and ileum was higher in Tianfu Meat Goose after force-feeding (*P* < 0.05).

### Comparison of Force-Feeding Influence on Digestive Enzyme Activity and Antioxidant Capacity in Small Intestine in 2 Goose Breeds

**Figure 3:**
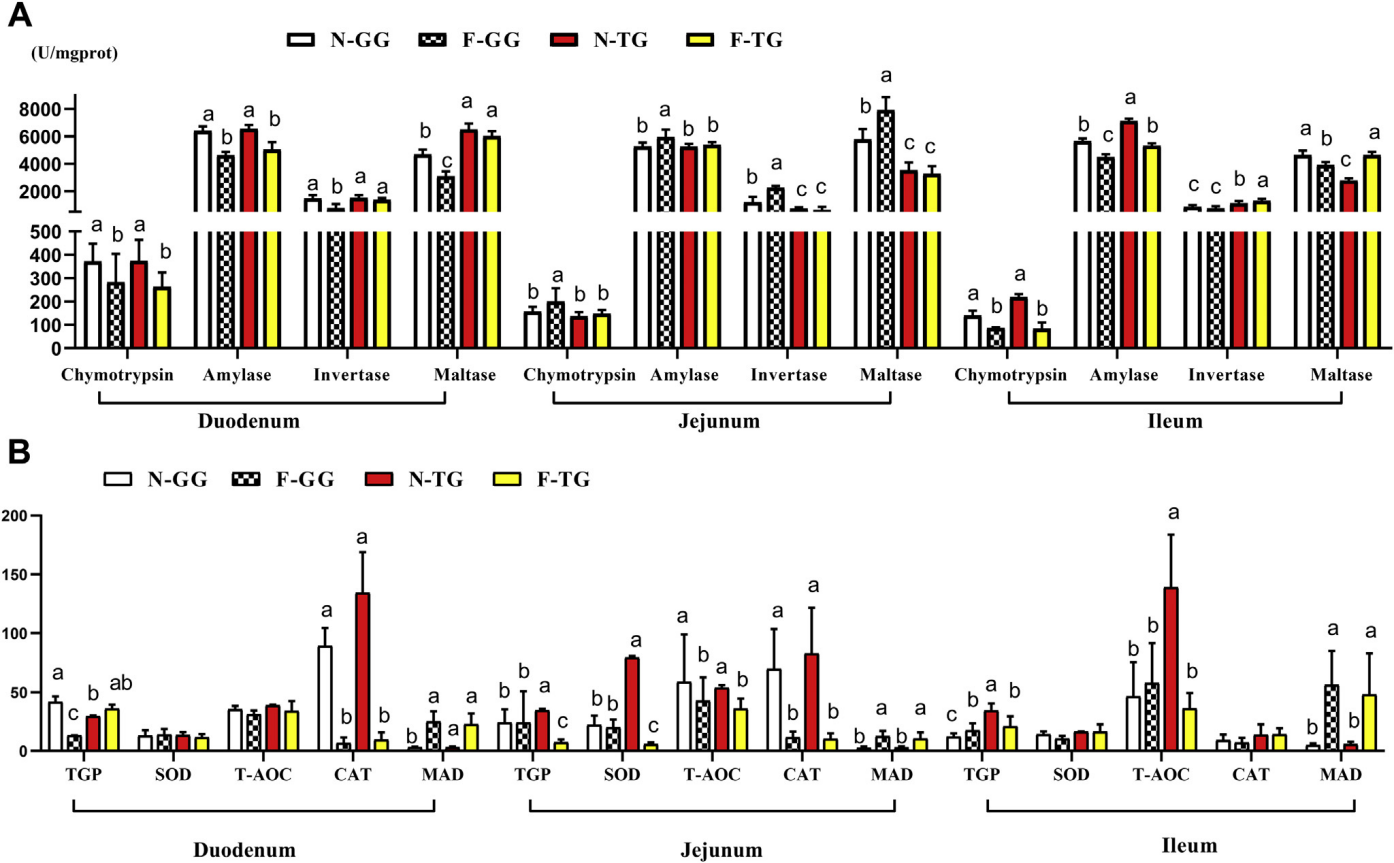
Comparison of force-feeding influence between 2 breeds on intestinal digestive enzyme activity and antioxidant performance. (A) Force-feeding influence on small intestinal digestive enzyme activity. (B) Force-feeding influence on small intestinal antioxidant performance. The unit of CAT is U/mg-prot, the unit of MAD is nmol/mg-prot, the unit of SOD is U/mg-prot, the unit of T-AOC is mmol/g-prot, the unit of TGP is mU/mg-prot. The experimental values are the means ± SD (n = 5). The different lowercase above the bars represent significant differences (*P* < 0.05). Abbreviations: CAT, catalase; F-GG, force-feeding group of Gang Goose; F-TG, force-feeding group of Tianfu Meat Goose; MAD, malonyldialdehyde; N-GG, normal-feeding group of Gang Goose; N-TG, normal-feeding group of Tianfu Meat Goose; SOD, superoxide dismutase; T-AOC, total antioxidant capacity; TGP, total glutathione peroxidase.

Compared with Gang Goose, the activity of maltase and invertase in duodenum and ileum was higher in Tianfu Meat Goose after force-feeding (*P* < 0.05) (Figure 3A). Figure 3B showed the influence of force-feeding on the intestinal antioxidant performance. Compared with Gang Goose, the activity of TGP and T-AOC of jejunum and ileum was higher in Tianfu Meat Goose before overfeeding, and SOD activity of ileum was higher in Tianfu Meat Goose after force-feeding (*P* < 0.05). Force-feeding increased the MAD contents and decreased the activity of CAT in both breeds (*P* < 0.05).

### Comparison of Force-Feeding Influence on Gut Flora in 2 Goose Breeds

**Figure 4:**
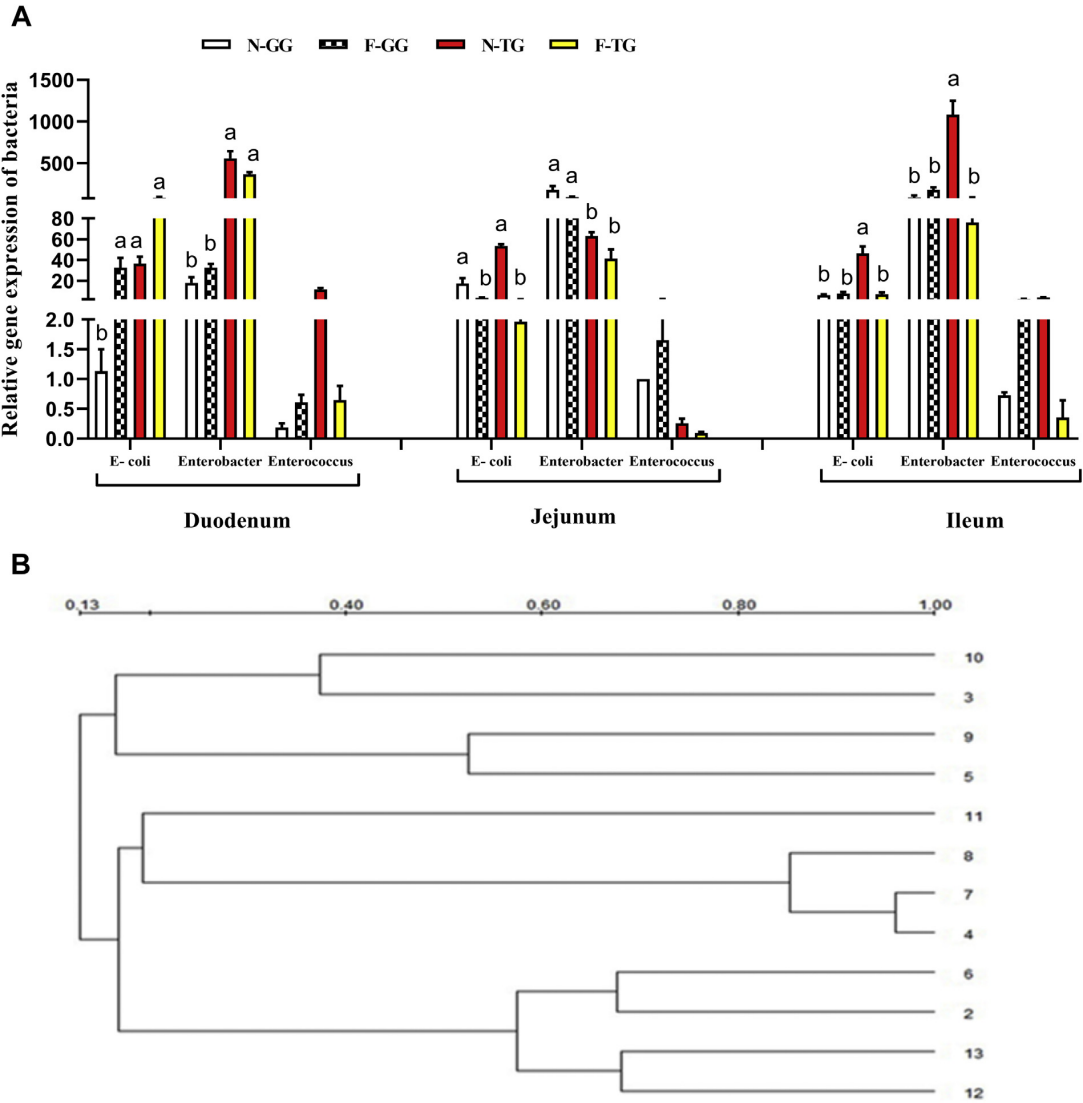
Comparison of force-feeding influence between 2 breeds on gut microbiota. (A) Relative gene expression level of bacteria in small intestine. (B) The dendrogram of ERIC-PCR in the intestinal microbiota. 2–5 represent ERIC-PCR results of the intestinal microbiota in duodenum section; 2: N-GG; 3: F-GG; 4: N-TG; 5: F-TG. 6–9 represent ERIC-PCR results of the intestinal microbiota in jejunum section; 6: N-GG; 7: F-GG; 8: N-TG; 9: F-TG; 10–13 represent ERIC-PCR results of the intestinal microbiota in ileum section; 10: N-GG; 11: F-GG; 12: N-TG; 13: F-TG. The experimental values are the means ± SD (n = 5). The different lowercase above the bars represent significant differences (*P* < 0.05). Abbreviations: *E-coli, Escherichia coli*; F-GG, force-feeding group of Gang Goose; F-TG, force-feeding group of Tianfu Meat Goose; N-GG, normal-feeding group of Gang Goose; N-TG, normal-feeding group of Tianfu Meat Goose.

As shown in Figure 4A, the overall distribution tendency of *Enterococcus*, *Enterobacter*, and *Escherichia coli* was similar in each part of the intestine (*Enterobacter* > *E. coli* > *Enterococcus*) in the 2 groups of Gang Goose and Tianfu Meat Goose. Before force-feeding, *Enterobacter* gene expression of Gang Goose was higher than that of Tianfu Meat Goose in jejunum and ileum (*P* < 0.05). There was no significant difference in the gene expression of *Enterococcus* between 2 breeds after force-feeding (*P* > 0.05). Compared with Gang Goose, the gene expression of *E. coli* decreased in the ileum of Tianfu Meat Goose after force-feeding (*P* < 0.05). The gene expression of *Enterobacter* in the jejunum of Gang Goose was higher than that of Tianfu Meat Goose after force-feeding (*P* < 0.05). Figure 4B showed the similarity of the ERIC-PCR map; the similar index between the microbiota in different intestinal segments varied from 0.13 to 1.00. Cluster analysis indicated that forced-feeding had an evident influence on the intestinal microbiota in both goose breeds. The number of amplified bands of intestinal microbiota in each intestinal segment of Gang Goose was decreased in an overfed group than that of the control group. In contrast, the number of amplified bands of intestinal microbiota in each intestinal segment of Tianfu Meat Goose was increased in the overfed group than that of the control group (Supplementary Figure 3).

## Discussion

The nutrients that the body needs for growth come from diet; these nutrients are digested in the gastrointestinal tract and absorbed into the body to ensure the normal growth and development of the body and the physiological functions. Thus, intestinal health and integrity play a crucial role in animal growth. Intestinal development promoted the nutrition digestion and absorption (Fang et al., 2014; Qin et al., 2019); laying hens increased the length of the small intestine to deal with the negative implications of rapid feed passage on the digestion and absorption of nutrients (Ege et al., 2019). Mitchell and Smith (1991) reported that the absolute intestinal weight and length showed positive correlation with growth rate in a broiler. In the current study, the intestinal weight and length increased after force-feeding, which showed that force-feeding promoted intestinal growth and development. In addition, the intestinal morphology is another indicator of intestinal health and integrity. A longer villi length and deeper crypts have been reported. This is indicative of decreased nutrient absorption, and the higher ratio of villus height to crypt depth reflects higher nutrient absorption capacity (Wang et al., 2018; Qin et al., 2019). The present trial showed that the villus height, the crypt depth decreased, and the ratio of villus height to crypt depth increased in the intestine after force-feeding. Meanwhile, the weight and length of intestinal increased after force-feeding, which showed that gut accelerated its own growth and development to adapt to the high-intensity digestion and absorption. These changes of intestinal morphology are in line with the results that the body weight and the liver weight increased, the fat deposited in the subcutaneous and abdomen after force-feeding. The villus height/crypt depth of Tianfu Meat Goose was higher than that of Gang Goose in the duodenum and ileum after force-feeding, which suggested that the intestinal absorption capacity of Tianfu Meat Goose was better than that of Gang Goose.

Nutrients in food are broken down into small molecular substances, such as monosaccharides, amino acids, and small peptides, by digestive enzymes. These small molecular substances are absorbed into the blood via the intestinal tract and participate in synthetic reaction. More and more reports showed that there is a positive correlation between animal growth performance and the activity of the intestine digestive enzyme (Yu et al., 2019; Jiang et al., 2020; Long et al., 2020a). After force-feeding, the waterfowl received high energy; therefore, the substrates for fatty acid synthesis (glucose) increased substantially in the liver. Meanwhile, the content of triglycerides (TG) produced in the liver far exceeded the transport capacity of apolipoproteins, and the fatty acid produced in the liver far exceeded the degraded capacity of fatty acid by *β*-oxidation, thus leading to the accumulation of lipids in the liver (Wei et al., 2020). Invertase and maltase are disaccharidase and can catalyze disaccharide coming from starch cracking into glucose. The activity of invertase and maltase of Tianfu Meat Goose was higher in the duodenum and ileum and lower in the jejunum than that of Gang Goose after force-feeding. The ileum in poultry has characteristic ileal digestion (Jamroz et al., 2002; Abdelfattah-Hassan and El-Ghazaly, 2019); the ileum is the main contributor to the enhanced capacity of nutrient digestion and absorption (Gu et al., 2020). In accordance with previous studies, the total length of duodenum and ileum (53.10 cm + 85.40 cm, 47.40 cm + 88.20 cm; Gang Goose and Tianfu Meat Goose, respectively) was longer than the length of the jejunum (73.90 cm, 75.90 cm; Gang Goose and Tianfu Meat Goose, respectively) in this current experiment after force-feeding, which suggested that the activity of disaccharidase (invertase and maltase) of Tianfu Meat Goose was higher than that of Gang Goose in the small intestine. As Figure 2B described, the ratio of villus height to crypt depth of Tianfu Meat Goose was higher than that of Gang Goose after force-feeding. Combined with the synergy action of the digestive enzyme activity and the ratio of villus height to crypt depth, the digestion–absorption capacity of Tianfu Meat Goose was higher than that of Gang Goose. Thereby, after force-feeding, more and more glucose was absorbed into the blood and synthesized TG in the liver of Tianfu Meat Goose, which caused heavier lipid deposition and larger liver weight.

Because of physical expansion caused by overfeeding, the intestinal wall thickness decreased after overfeeding in this experiment (Figure 2B), which means that intestine barrier permeability increased. Food was digested and decomposed in the intestinal tract; this process produced a variety of oxidizing factors (Qiu et al., 2020). Increased permeability also increased the amount of peroxides in the blood, increasing the oxidative stress of the body (Li et al., 2019), and induced an inflammatory response (Zhu et al., 2020). In the present study, the activities of TGP, SOD, CAT, and T-AOC decreased and the MAD content increased in the intestine after force-feeding, which indicated that overfeeding caused oxidative stress in the intestine tract, and the intestine tract and body faced the challenge of oxidative stress in overfeeding process. The antioxidant substances in the intestinal tract can eliminate the peroxide in the intestinal tract and protect the intestinal mucosa (Lin et al., 2016; Qiu et al., 2020). Similarly, the gut antioxidant performance will influence the activity of digestive enzymes (Long et al., 2020b; Mountzouris et al., 2020). The antioxidant enzymes such as SOD and CAT are indispensable key factors against oxidative stress that have the capacity to decompose superoxide and eliminate free radicals by using a chain reaction mechanism (Shirani et al., 2019). Before overfeeding, the activity of TGP and T-AOC in jejunum and ileum of Tianfu Meat Goose was higher than that of Gang Goose; and SOD activity in the ileum of Tianfu Meat Goose was higher than that of Gang Goose after overfeeding, and ileum is the uppermost segments of intestine, which indicated that Tianfu Meat Goose possessed stronger antioxidant capacity in the small intestine.

The intestine is the place where the nutrients are digested and absorbed and also where the intestinal microbes are colonized. A study of Langdes Goose showed that the richness and diversity of the bacterial communities decreased in the ileum and cecum after overfeeding (Tang et al., 2018). It was reported that the proliferation of *C. perfringens* eliminated the absorptive villi and resulted in the crypts between them to deepen, thus making the intestinal lining nonabsorptive, thick, and lumpy (Zanu et al., 2020). In addition, when the number of harmful intestinal microbes is increased, their metabolites will lead to changes in intestinal structure and permeability. The increased permeability also increases the amount of microbes, their endotoxin or lipopolysaccharide, and harmful metabolites entering the intestine, causing a systemic immune response, inflammation, and changes in the functions of other organs and tissues (Luci et al., 2019). Therefore, the stabilization of intestinal microflora is critical to intestinal health, barrier function, and nutrient absorption (Mohebodini et al., 2019). *Enterobacter* is involved in fat accumulation and lipid metabolism (Ferreira Barletta et al., 2016; Priyadarsini et al., 2020). *Enterococcus faecium* belongs to *Lactobacillus*, and it is a normal beneficial bacterium in the gastrointestinal tract of mammals (Zhao et al., 2013; Castaneda et al., 2020). The *E. coli* causes immunological stress by damaging the intestine of poultry and produces lipopolysaccharide, which can affect the physiological and pathological processes of poultry and interfere with their normal function (Munyaka et al., 2012). Therefore, in this study, *Enterococcus*, *E. coli*, and *Enterobacteria* were selected for analysis. In this study, the gene expression of *Enterobacter* in Gang Goose was higher in the jejunum and ileum and lower in the duodenum than that of Tianfu Meat Goose before overfeeding; the gene expression of *Enterobacter* in Gang Goose was higher in the jejunum and lower in the duodenum than that of Tianfu Meat Goose after force-feeding; as said above, the total length of jejunum and ileum was longer than the length of the duodenum. These results suggested that the number of *Enterobacter* in the small intestine of Gang Goose was bigger than that of Tianfu Meat Goose. The subcutaneous adipose tissue weight of Gang Goose was higher than that of Tianfu Meat Goose after overfeeding. It was consistent with a new research result that *Enterobacter cloacae* administration induced the subcutaneous fat accumulation in the high-fat diet–fed mice (Keskitalo et al., 2018). Pathogenic bacteria entering the intestinal tract reduced the species and number of intestinal microorganisms by producing toxins and harmful substances and reduced the diversity of intestinal flora (Abdelhamid et al., 2020; Siddiqui et al., 2020). In the current experiment, force-feeding influence decreased the gene expression of *E. coli* in Tianfu Meat Goose after overfeeding. In addition, ERIC-PCR results showed that overfeeding decreased the band number of the intestinal bacteria in each intestinal segment of Gang Goose and increased the band number of the intestinal bacteria in each intestinal segment of Tianfu Meat Goose, and the number of ERIC-PCR bands reflected the microbial diversity (Ramees et al., 2014; Peng et al., 2014; Guo et al., 2016; Khan et al., 2018). Q-PCR and ERIC-PCR results indicated that the small intestinal microbial diversity of Tianfu Meat Goose was higher after overfeeding. The increasing intestinal microbial diversity increased intestinal homeostasis and resistance to pathogens (Mountzouris et al., 2015; Kim et al., 2020; Rodrigues et al., 2020). Thereby, intestinal microflora stabilization of Tianfu Meat Goose was higher than that of Gang Goose under force-feeding. As discussed above, Tianfu Meat Goose also had a stronger antioxidant capacity in the small intestine; therefore, Tianfu Meat Goose possessed better intestinal homeostasis and stronger tolerance for force-feeding than Gang Goose. In brief, some breeds of the goose are selectively bred specifically for *foie gras* production. For example, Landes Goose has a greater *foie gras* performance than other goose breeds or other palmipede breeds. Therefore, it is assumed that the digestion–absorption capacity, intestinal homeostasis, and gut flora stability response to force-feeding are probably maximized in Landes Goose, which makes Landes Goose more adaptive to lipid deposition in the liver than other goose breeds.

## Conclusion

Different goose breeds have different gut physiology characteristics and gut microbiota; conversely, the gut physiology characteristic and gut microbiota contribute to shaping different production performances. In this study, after geese were force-fed, Gang Goose showed better fat storage capacity in subcutaneous adipose tissue. Responded to excessive diet intake, Tianfu Meat Goose showed better digestion–absorption capacity, intestine antioxidant capacity, gut microbiota diversity, and gut microbiota stability, thus, showed better performance in *foie gras*. So, Tianfu Meat Goose was the better breed for *foie gras* production. This study not only provided a reference for the development and utilization of breed resource for *foie gras* production but also provided a new method to induce the fat accumulation in different tissue in force-feeding, such as gut flora diversity and intestinal environment are changed via supplementing probiotics in overfeeding diet, which may induce more lipids deposition in liver and fewer lipids deposition in adipose tissue, and then, improve the yield and quality of *foie gras*.
