# Supplementary material for: Comparison of overfeeding effects on gut physiology and microbiota in two goose breeds
Source: Poult Sci. 2020 Dec 24;100(3):100960. doi: 10.1016/j.psj.2020.12.057 (PMC7936201; doi:10.1016/j.psj.2020.12.057)
Supplement: Supplementary Material [file mmc1.docx]

**Suplementary**


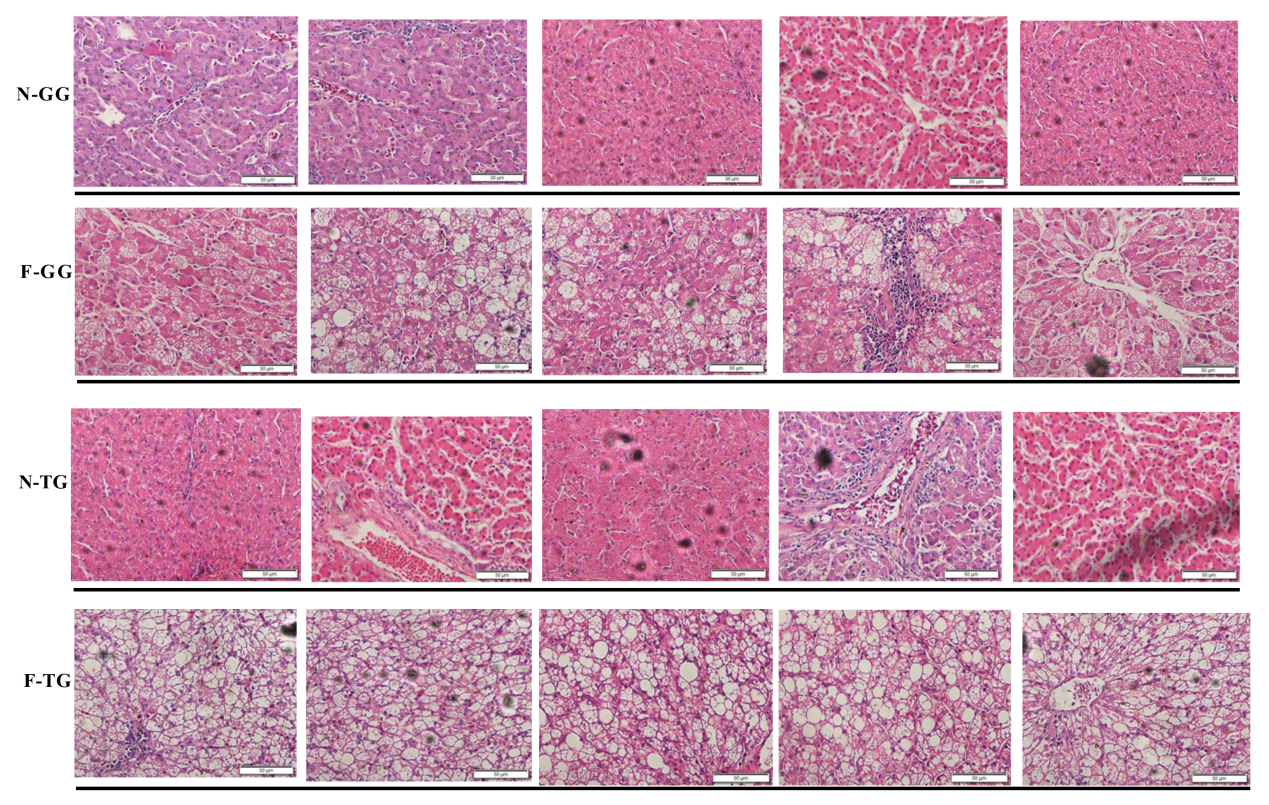


**S-Figure 1.** Comparison of liver histology between two goose breeds; 20×; (n=5).

N-GG = Normal-feeding group of Gang Goose; F-GG = Force-feeding group of Gang Goose;

N-TG = Normal-feeding group of Tianfu Meat Goose; F-TG = Force-feeding group of Tianfu Meat Goose.


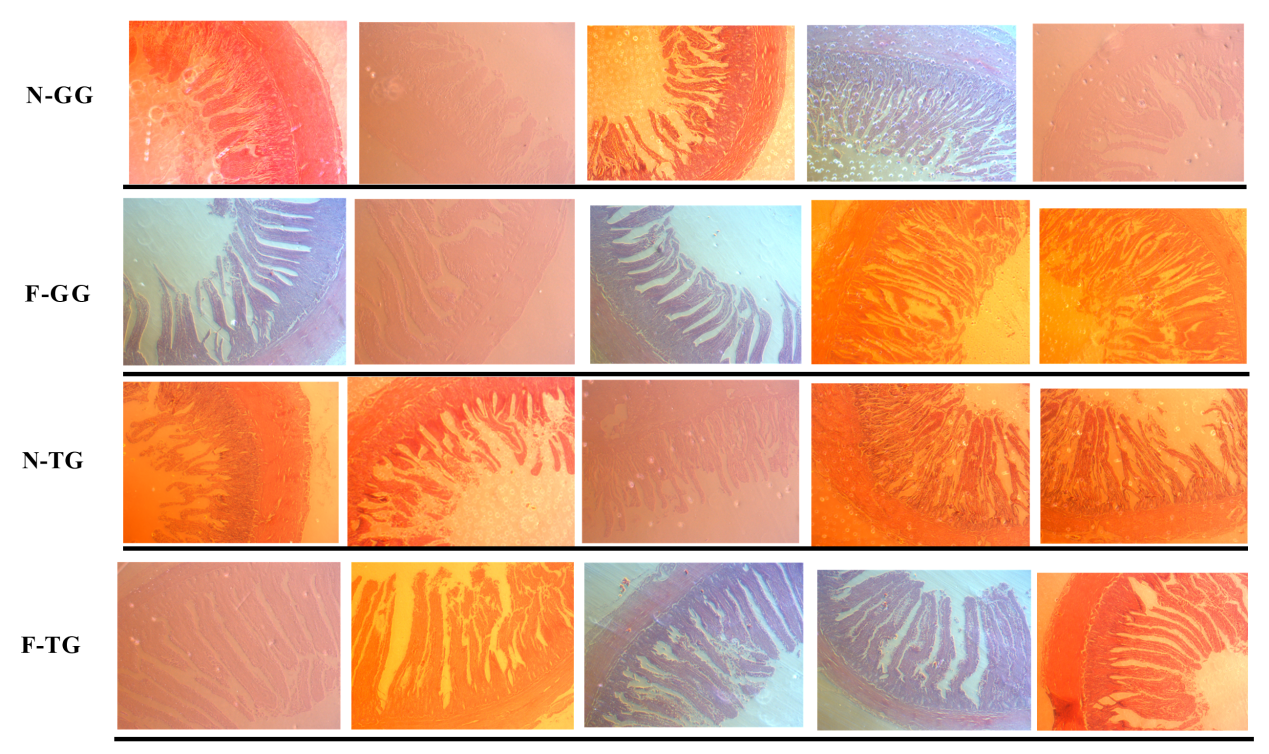


**S-Figure 2.** Comparison of small intestinal histology between two goose breeds in duodenum section. 20×; (n=5). N-GG = Normal-feeding group of Gang Goose; F-GG = Force-feeding group of Gang Goose; N-TG = Normal-feeding group of Tianfu Meat Goose; F-TG = Force-feeding group of Tianfu Meat Goose.


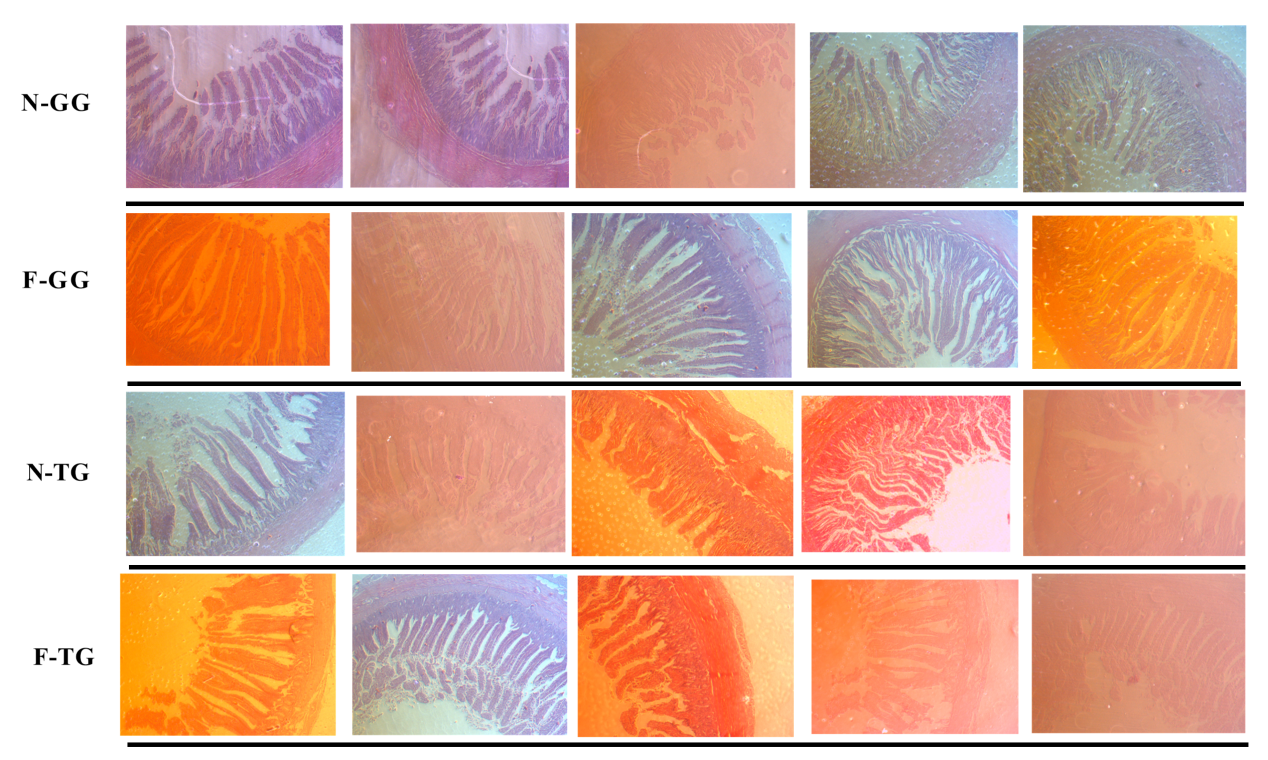


**S-Figure 3.** Comparison of small intestinal histology between two goose breeds in jejunum section. 20×; (n=5). N-GG = Normal-feeding group of Gang Goose; F-GG = Force-feeding group of Gang Goose; N-TG = Normal-feeding group of Tianfu Meat Goose; F-TG = Force-feeding group of Tianfu Meat Goose.


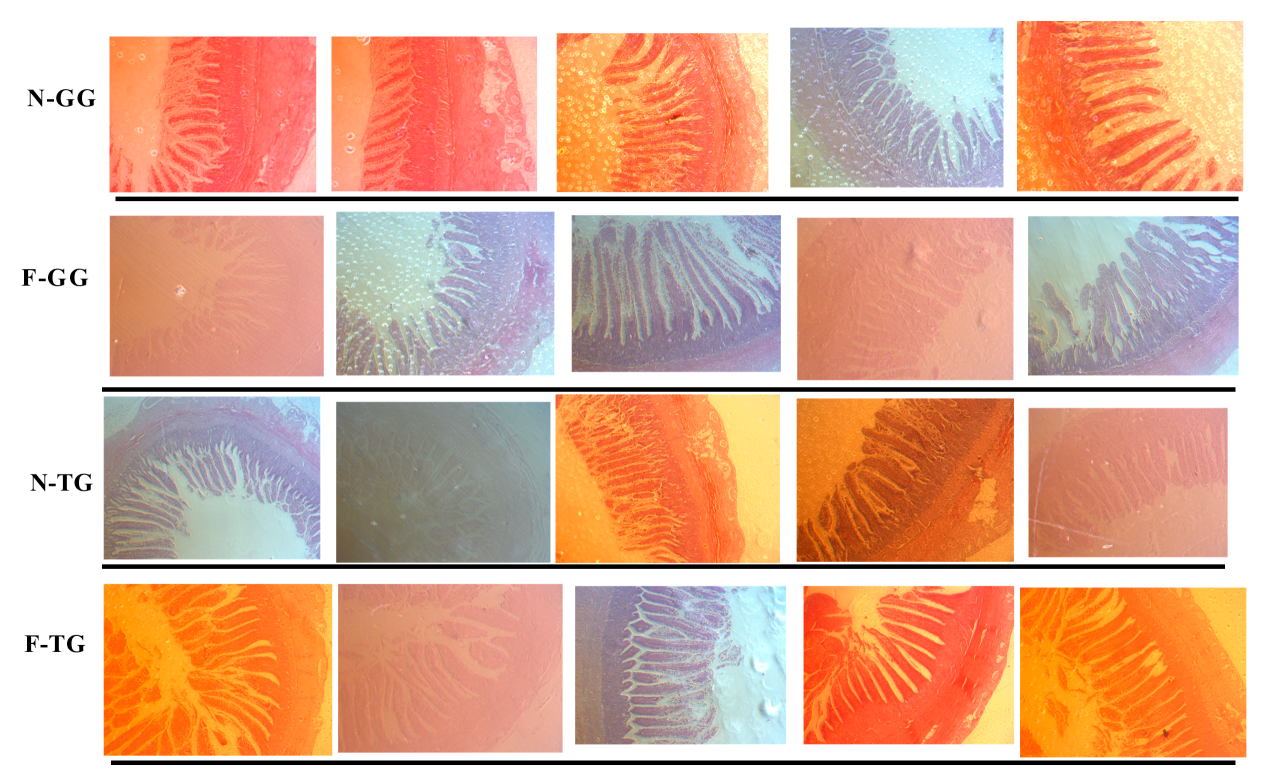


**S-Figure 4.** Comparison of small intestinal histology between two goose breeds in ileum section. 20×; (n=5). N-GG = Normal-feeding group of Gang Goose; F-GG = Force-feeding group of Gang Goose; N-TG = Normal-feeding group of Tianfu Meat Goose; F-TG = Force-feeding group of Tianfu Meat Goose.


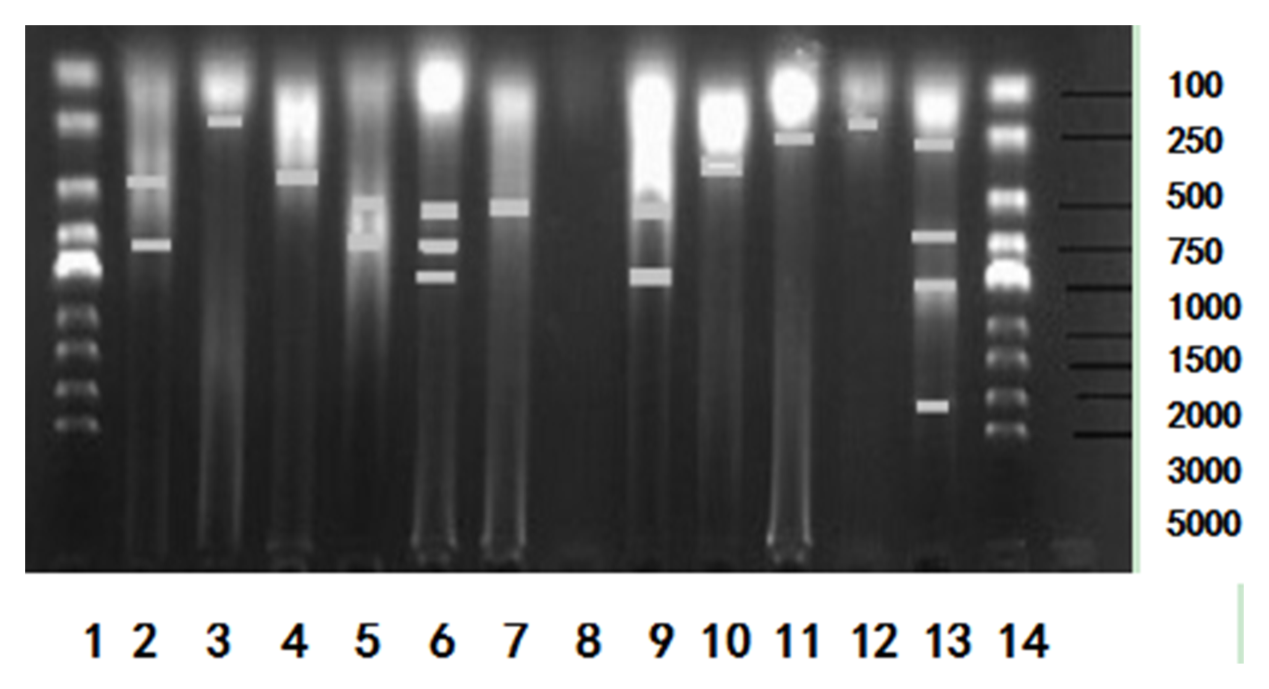


**S-Figure 3.** ERIC-PCR results of the small intestinal microbiota. 1and 14: DNA marker DL5000 ; 2-5 represent ERIC-PCR results of the intestinal microbiota in duodenum section; 2: N-GG; 3: F-GG; 4: N-TG; 5: F-TG. 6-9 represent ERIC-PCR results of the intestinal microbiota in jejunum section; 6: N-GG; 7: F-GG; 8: N-TG; 9: F-TG; 10-13 represent ERIC-PCR results of the intestinal microbiota in ileum section; 10: N-GG; 11: F-GG; 12: N-TG; 13: F-TG. N-GG = Normal-feeding group of Gang Goose; F-GG = Force-feeding group of Gang Goose; N-TG = Normal-feeding group of Tianfu Meat Goose; F-TG = Force-feeding group of Tianfu Meat Goose.

**S-Table 1.** Primers of q- PCR for three bacteria in small intestine

| Gene Name | Upstream (5′-3′) | Downstream (5′-3′) | Product size (bp) |
| --- | --- | --- | --- |
| *18S* | TTGGTGGAGCGATTTGTC | ATCTCGGGTGGCTGAACG | 129 |
| *β-actin* | CAACGAGCGGTTCAGGTGT | TGGAGTTGAAGGTGGTCTCG | 92 |
| *Escherichia coli* | TACCCGCAGAAGAAGCACC | CGCATTTCACCGCTACACC | 215 |
| *Enterobacter* | CATTGACGTTACCCGCAGAAGAAGC | CTCTACGAGACTCAAGCTTGC | 195 |
| *Enterococcus* | CCCTTATTGTTAGTTGCCATATT | CTCTACGAGACTCAAGCTTGC | 129 |
